# Eukaryotic Cell Toxicity and HSA Binding of [Ru(Me_4_phen)(bb_7_)]^2+^ and the Effect of Encapsulation in Cucurbit[10]uril

**DOI:** 10.3389/fchem.2018.00595

**Published:** 2018-11-30

**Authors:** Biyun Sun, Ian F. Musgrave, Anthony I. Day, Kirsten Heimann, F. Richard Keene, J. Grant Collins

**Affiliations:** ^1^School of Physical, Environmental and Mathematical Sciences, University of New South Wales, Australian Defence Force Academy, Canberra, ACT, Australia; ^2^Discipline of Pharmacology, Adelaide Medical School, University of Adelaide, Adelaide, SA, Australia; ^3^College of Medicine and Public Health, Flinders University, Adelaide, SA, Australia; ^4^College of Science and Engineering, James Cook University, Townsville, QLD, Australia; ^5^Department of Chemistry, School of Physical Sciences, University of Adelaide, Adelaide, SA, Australia; ^6^Australian Institute of Tropical Health and Medicine/Centre for Molecular Therapeutics, James Cook University, Townsville, QLD, Australia

**Keywords:** ruthenium complexes, cytotoxicity, HSA binding, cucurbit[10]uril, supramolecular chemistry

## Abstract

The toxicity (IC_50_) of a series of mononuclear ruthenium complexes containing bis[4(4′-methyl-2,2′-bipyridyl)]-1,*n*-alkane (bb_n_) as a tetradentate ligand against three eukaryotic cell lines—BHK (baby hamster kidney), Caco-2 (heterogeneous human epithelial colorectal adenocarcinoma) and Hep-G2 (liver carcinoma)—have been determined. The results demonstrate that *cis*-α-[Ru(Me_4_phen)(bb_7_)]^2+^ (designated as α-Me_4_phen-bb_7_, where Me_4_phen = 3,4,7,8-tetramethyl-1,10-phenanthroline) showed little toxicity toward the three cell lines, and was considerably less toxic than *cis*-α-[Ru(phen)(bb_12_)]^2+^ (α-phen-bb_12_) and the dinuclear complex [{Ru(phen)_2_}_2_{μ-bb_12_}]^4+^. Fluorescence spectroscopy was used to study the binding of the ruthenium complexes with human serum albumin (HSA). The binding of α-Me_4_phen-bb_7_ to the macrocyclic host molecule cucurbit[10]uril (Q[10]) was examined by NMR spectroscopy. Large upfield ^1^H NMR chemical shift changes observed for the methylene protons in the bb_7_ ligand upon addition of Q[10], coupled with the observation of several intermolecular ROEs in ROESY spectra, indicated that α-Me_4_phen-bb_7_ bound Q[10] with the bb_7_ methylene carbons within the cavity and the metal center positioned outside one of the portals. Simple molecular modeling confirmed the feasibility of the binding model. An α-Me_4_phen-bb_7_-Q[10] binding constant of 9.9 ± 0.2 × 10^6^ M^−1^ was determined by luminescence spectroscopy. Q[10]-encapsulation decreased the toxicity of α-Me_4_phen-bb_7_ against the three eukaryotic cell lines and increased the binding affinity of the ruthenium complex for HSA. Confocal microscopy experiments indicated that the level of accumulation of α-Me_4_phen-7 in BHK cells is not significantly affected by Q[10]-encapsulation. Taken together, the combined results suggest that α-Me_4_phen-7 could be a good candidate as a new antimicrobial agent, and Q[10]-encapsulation could be a method to improve the pharmacokinetics of the ruthenium complex.

## Introduction

Due to the increasing resistance of bacteria, particularly Gram-negative species, to the range of drugs currently in clinical use there is significant interest in developing new antimicrobial agents (Boucher et al., 2009). While there is an on-going effort to produce new antimicrobial drugs based upon analogs of known scaffolds, e.g., β-lactam antibiotics (Boucher et al., 2009), there is an increasing focus on the use of metal-based compounds (Richards et al., 2009; Neelakantan et al., 2010; Ng et al., 2013; Pandrala et al., 2013). In particular, and reflecting a greater recognition of their therapeutic potential (Bergamo and Sava, 2007; Moucheron, 2009; Süss-Fink, 2010; Gill and Thomas, 2012; Li et al., 2018), has been the growing development of ruthenium complexes as potential antimicrobial agents (Li et al., 2015a; Southam et al., 2017; Mital and Ziora, 2018). Dwyer and co-workers demonstrated more than 60 years ago the activity of mononuclear polypyridylruthenium(II) complexes against both Gram-positive and Gram-negative bacteria (Dwyer et al., 1952, 1969). However, it has only been over the last 10 years that there has been renewed and widespread interest in examining the antimicrobial properties of a broad range of ruthenium complexes (Kumar et al., 2009, 2016; Bolhuis et al., 2011; Li et al., 2011, 2013a, 2014; Shobha Devi et al., 2013; Gorle et al., 2016).

**Figure 1:**
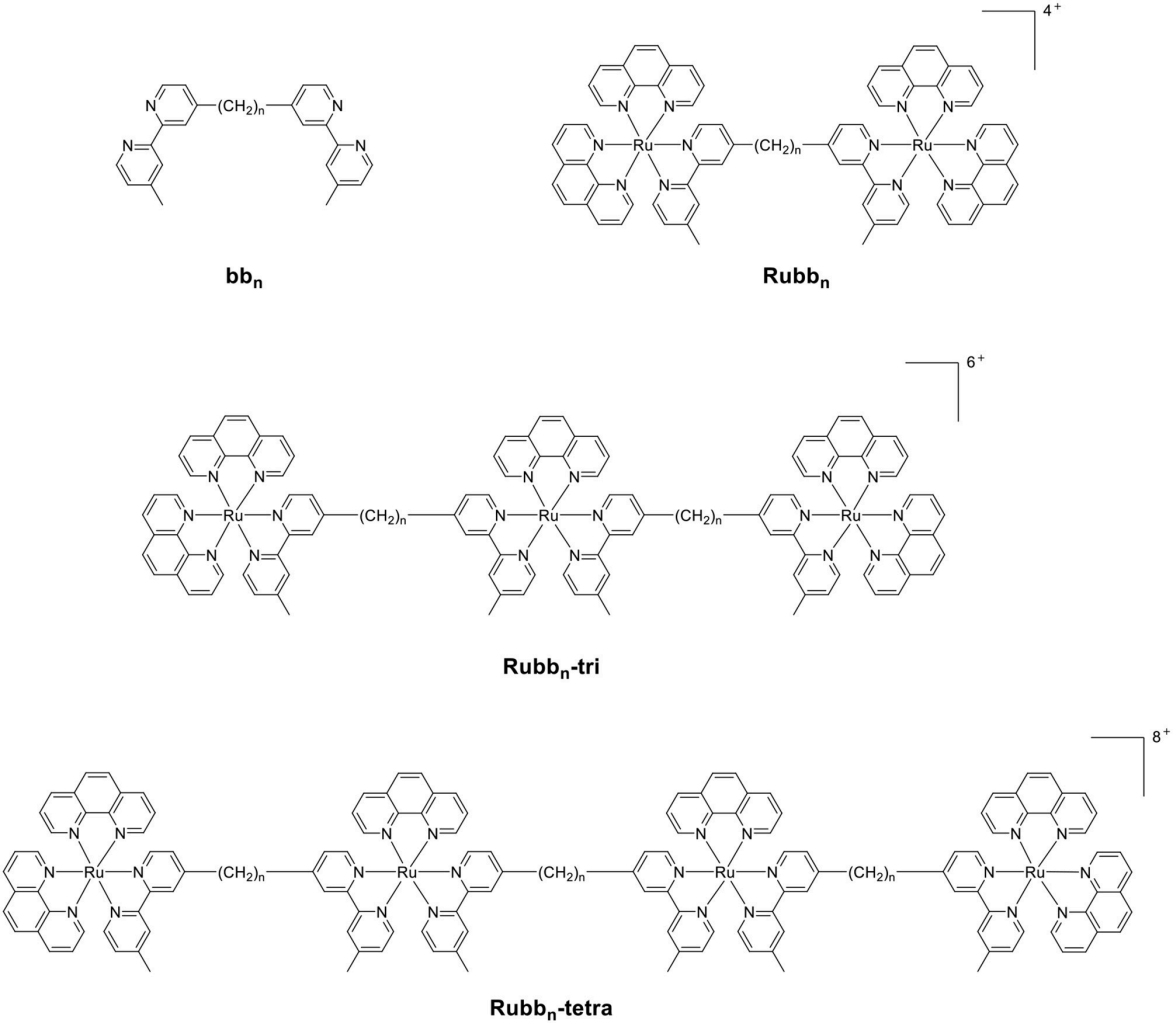
The structure of the bb_n_ ligand and the associated oligonuclear ruthenium complexes.

Previous studies from our group have examined the antimicrobial properties of di-, tri- and tetra-nuclear polypyridylruthenium(II) complexes in which the metal centers are linked by the bis[4(4′-methyl-2,2′-bipyridyl)]-1,*n*-alkane ligand (“bb_n_”; see Figure 1) (Li et al., 2011, 2016; Gorle et al., 2014). These oligonuclear ruthenium complexes showed excellent activity against Gram-positive bacteria, and maintained the activity against current drug-resistant strains such as methicillin-resistant *Staphylococcus aureus* (MRSA) and vancomycin-resistant *enterococci* (VRE) (Gorle et al., 2016). However, the oligonuclear complexes showed variable activity to Gram-negative strains, with a number of species being essentially resistant to the ruthenium complexes (Gorle et al., 2016).

**Figure 2:**
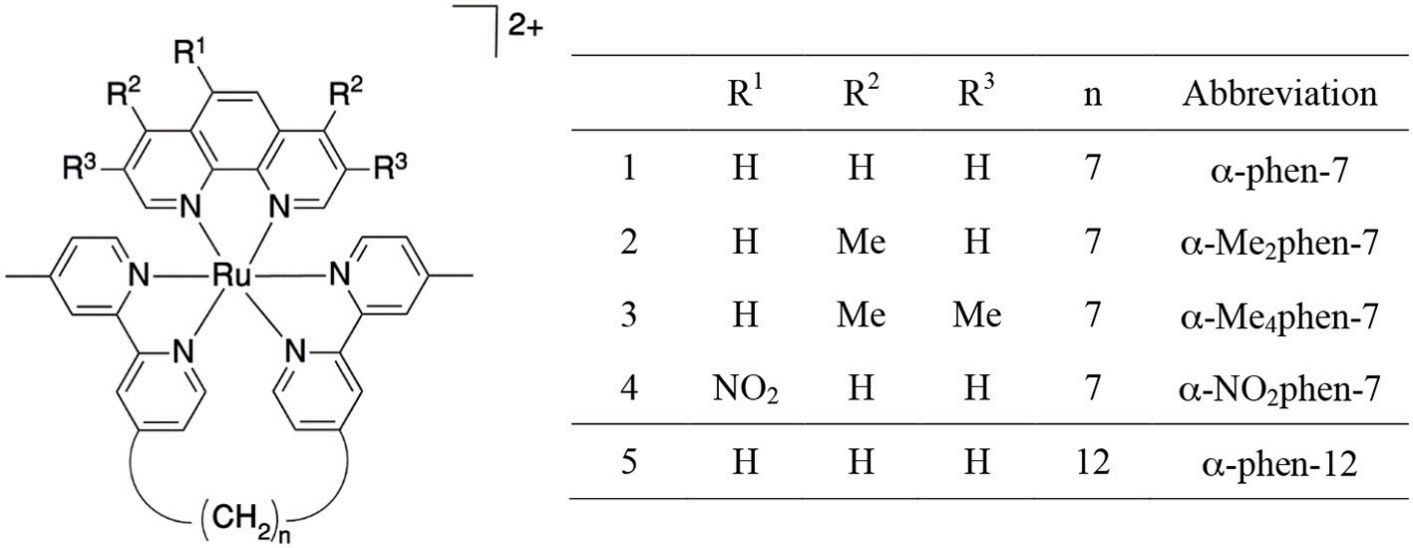
Structure and abbreviations for the *cis*-α-[Ru(phen′)(bb_n_)]^2+^ complexes {*n* = 7; phen′ = 1,10-phenanthroline (phen); 4,7-dimethyl-1,10-phenanthroline (Me_2_phen); 3,4,7,8-tetramethyl-1,10-phenanthroline (Me_4_phen); and 5-nitro-1,10-phenanthroline (NO_2_phen), *n* = 12; phen′ = 1,10-phenanthroline}.

As the toxicity to both bacterial and eukaryotic cells increased with the number of ruthenium centers in the oligonuclear complex (Li et al., 2012, 2015b), we sought to examine mononuclear complexes that contained the bb_n_ moiety as a tetradentate ligand—[Ru(phen′)(bb_n_)]^2+^ complexes (where phen′ = 1,10-phenanthroline and a variety of its derivatives, see Figure 2) (Gorle et al., 2015; Sun et al., 2018). Although some of these mononuclear complexes—particularly *cis*-α-[Ru(Me_4_phen)(bb_7_)]^2+^ (Me_4_phen = 3,4,7,8-tetramethyl-1,10-phenanthroline), designated as α-Me_4_phen-7; and *cis*-α-[Ru(phen)(bb_12_)]^2+^, designated as α-phen-12—showed good and uniform activity against both Gram-positive and Gram-negative species, they were slightly less active than the oligonuclear complexes to most bacteria (Sun et al., 2018). However, while the toxicity toward eukaryotic cells has been established for the oligonuclear complexes (Li et al., 2015b), the corresponding data for the mononuclear complexes have yet to be reported.

Another important factor governing the clinical potential of a new compound is its ability to bind serum proteins (Kratochwil et al., 2002; Bohnert and Gan, 2013; Liu et al., 2014). Blood serum protein binding can affect the distribution and clearance of a drug in several ways: only the free (non-protein bound) drug can act as an antimicrobial agent; alternatively, non-protein bound small drugs are cleared more rapidly from the bloodstream. Again, while the binding of the oligonuclear complexes to human serum albumin (HSA)–the most abundant serum protein (0.6 mM in healthy humans)–has been determined for the oligonuclear complexes (Li et al., 2013b), it is yet to be reported for the mononuclear complexes.

**Figure 3:**
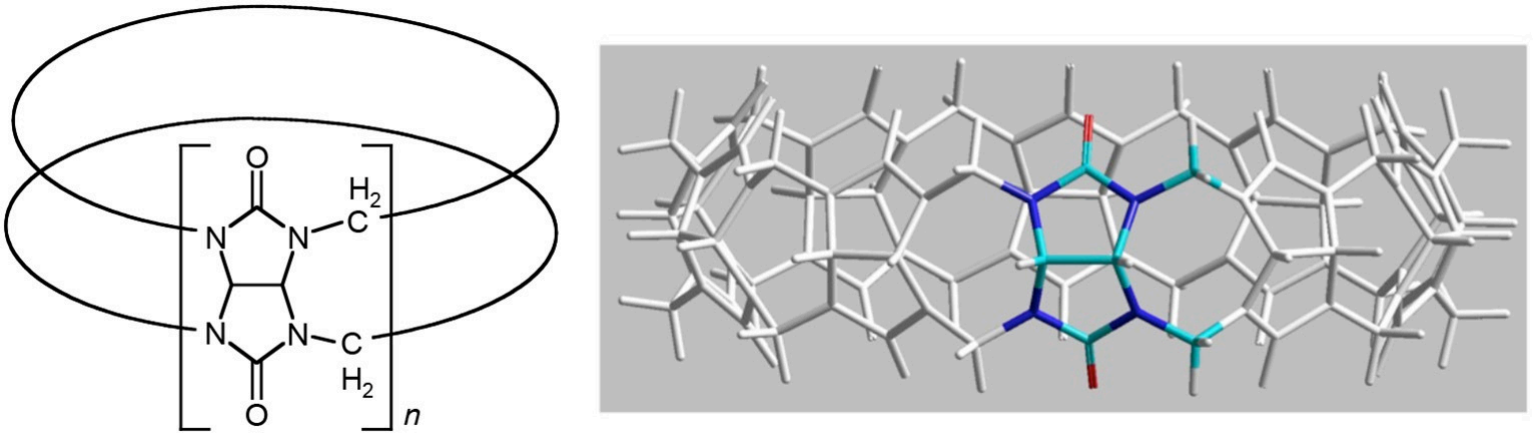
Two representations of the structure of cucurbit[*n*]uril.

In the present study, we have determined the toxicity of the mononuclear [Ru(phen′)(bb_n_)]^2+^ complexes toward a panel of eukaryotic cells and examined their HSA binding ability. Furthermore, as encapsulation in cucurbit[*n*]urils {macrocyclic host compounds composed of *n* glycoluril monomeric units, Q[*n*]–see Figure 3 (Lagona et al., 2005; Kim et al., 2007; Isaacs, 2009)} can potentially decrease the toxicity of a drug and modulate the drugs' ability to bind serum proteins, (Wheate et al., 2004; Jeon et al., 2005; Li et al., 2013b) we have also examined the ability of α-Me_4_phen-7 to form an inclusion complex with Q[10] and determined the subsequent effect on the toxicity of the ruthenium complex toward eukaryotic cells and its ability to bind HSA.

## Experimental

### Materials

The ruthenium complexes used in this study {*cis*-α-[Ru(phen)(bb_7_)]^2+^ (designated as α-phen-bb_7_), *cis*-α-[Ru(Me_2_phen)(bb_7_)]^2+^ (α-Me_2_phen-bb_7_), *cis*-α-[Ru(Me_4_-phen)(bb_7_)]^2+^ (α-Me_4_phen-bb_7_), *cis*-α-[Ru(NO_2_phen)(bb_7_)]^2+^ (α-NO_2_phen-bb_7_), *cis*-α-[Ru(phen)(bb_12_)]^2+^ (α-phen-bb_12_), [Ru(Me_4_phen)_3_]^2+^ and Rubb_12_} and Q[10] were synthesized as previously described (Liu et al., 2005; Pisani et al., 2010; Gorle et al., 2015; Sun et al., 2018).

### Toxicity

The toxicities of the ruthenium complexes were assayed against three eukaryotic cell lines: BHK (baby hamster kidney); Caco-2 (heterogeneous human epithelial colorectal adenocarcinoma); and Hep-G2 (liver carcinoma). All cell lines were cultured in 75 mL culture flasks in DMEM culture media (Dulbecco's Modified Eagle's Medium, Gibco, Thermo Fisher, UK) supplemented with 10% fetal bovine serum (Gibco, Thermo Fisher, UK), 1% MEM Non-Essential Amino Acid (Sigma, UK) and 1% penicillin-streptomycin (Sigma) at 37°C in an atmosphere of 5% humidified CO_2_. Cells used in the study were in the logarithmic growth phase, and unless stated were grown to 70% confluence, and then trypsinized with 0.5% trypsin-EDTA (Gibco, Canada) for detachment and use in the assays.

Half-maximal inhibitory concentrations (IC_50_) of the ruthenium complexes against BHK, Caco-2 and HepG2 cell lines were performed using the mitochondrial-dependent reduction of 3-(3,4-dimethylthiazol-2-yl)-5-diphenyl tetrazolium bromide (MTT) to formazan as previously described (Sundaraneedi et al., 2017). The cells were cultured in 96-well microtiter plates containing the respective media to a cell density of 3,000 per well at 37°C in an atmosphere of air containing 5% CO_2_. Cell viability was assessed after continuous exposure to various concentrations of drugs (ranging from 0.5 to 400 μM) for 24 and 48 h. Cisplatin served as a treatment evaluation standard. Ruthenium complex stocks were made to the required concentration in sterile Milli-Q water, and cisplatin solutions were made to the required concentration in culture medium before the treatment. The amount of MTT reduced to formazan within the cells was quantified by measuring the absorbance at λ = 570 nm using a Fluostar Galaxy Microplate Reader. The average values presented are based on at least three independent experiments. The IC_50_ values were determined using GraphPad Prism 7.0 (GraphPad Software, San Diego, USA).

### HSA binding

All solutions used in the protein binding studies were dissolved in a 0.1 M sodium phosphate buffer (pH = 7.4). Protein solutions (5 μM) were titrated with the ruthenium complexes from 2 mM stock solutions, from a metal complex/protein ratio of 0.0 to 8.0. The maximum fluorescence for HSA (Sigma-Aldrich) was observed at λ = 345 nm after excitation at λ = 280 nm. All experiments were carried out in triplicate, with results presented as mean and standard deviation.

### Encapsulation by Q[10]

#### NMR

^1^H NMR titrations were carried out by the addition of solid Q[10] (0.4 mg) into a solution of α-Me_4_phen-bb_7_ (2 mM, 600 μL) to a 1:1 Q[10]:α-Me_4_phen-bb_7_ ratio. ROESY NMR experiments for the free and Q[10]-encapsulated α-Me_4_phen-bb_7_ were conducted with 2048 data points in t2 for 256 t1 values, with a pulse repetition delay set to 1.7 s and mixing times of 350 ms.

#### Molecular modeling

The encapsulation of α-Me_4_phen-7 in the Q[10] cavity was examined by molecular modeling using the *HyperChem* (version 8, HyperCube, Inc.) program. The bb_7_ alkyl chain was positioned deep within the Q[10] cavity and the ruthenium metal center positioned at one of the portals in a manner consistent with the NMR results. The Q[10]-encapsulated α-Me_4_phen-7 was then optimized using the Amber99 molecular mechanics forcefield without explicitly treating water molecules (i.e., “*in vacuo*”). Successive geometry optimizations using slightly different starting conformations and different minimization algorithms produced similar results.

#### Luminescence titration

Spectra were recorded on a Horiba Scientific FluoroMax-4 fluorescence spectrophotometer at room temperature in a 1 cm quartz cuvette. Samples were excited at λ = 435 nm and emissions were recorded in the λ = 510–800 nm range with slit widths of 5 nm in increments of 1 nm. All luminescence spectroscopy samples were prepared in Milli-Q water. Luminescence titrations were carried out by titration of 20 μL aliquots of Q[10] (12.5 μM) stock solution into a α-Me_4_phen-7 (0.675 μM; 2,950 μL) solution to a 1.5:1 ratio. The sample was then sparged with argon to remove any oxygen present and allowed to equilibrate for 3 min before measurements were taken.

The binding constant (*K*_*b*_) for α-Me_4_phen-7 binding to Q[10] was calculated using Equation 1 (Pisani et al., 2010):

(1) $$\begin{array}{l} K_{b}=\frac{{\left[MC\right]}_{b}}{{\left[MC\right]}_{f}\times {\left[Q10\right]}_{f}} \end{array}$$

where [MC]_f_ and [Q10]_f_ are the concentrations of free α-Me_4_phen-7 and Q[10], respectively, at each titration point. [MC]_b_ was determined using Equation 2:

(2) $$\begin{array}{l} {\left[MC\right]}_{b}=\frac{{\left[MC\right]}_{t}}{1-\left(\frac{\delta_{obs}-\delta_{b}}{\delta_{f}\left(x\right)+\delta_{b}\left(x\right)}\right)} \end{array}$$

where [MC]_t_ is the concentration of metal complex in the sample, δ_obs_ is the luminescence intensity maxima observed at each titration point, δ_b_ is the luminescence intensity maximum of the bound metal complex, δ_f_ is the luminescence intensity maximum of the free metal complex and x is the ratio of bound/free metal complex at each titration point. The binding constant at each point was calculated and averaged to give an approximate binding constant.

#### Confocal microscopy

The trypsinized BHK cells were seeded on sterile, poly L-lysine coated, coverslips in a 24-well plate. The ruthenium complex was applied to the cells in growth media to make the desired concentration (25 μM) and incubated at 37°C with 5% CO_2_ for 1 h. Following the incubation, 100 nM Mitotracker® Green FM (Invitrogen) was added for mitochondrial staining. Staining was carried out in DMEM medium under standard cultivation conditions as per the manufacturer's instructions. Following staining, the coverslips were gently rinsed with phosphate buffer solution (PBS, pH = 7.1) prior to confocal laser scanning microscopy.

The cellular accumulation of the ruthenium complex was determined using a laser scanning confocal microscope (FV3000, Olympus). Samples were viewed under 60× silicone immersion using the following excitation (λ_ex_) and emission (λ_em_) wavelengths. Ruthenium complex (λ_ex_ = 450 nm, λ_em_ = 630 nm) and Mitotracker Green FM (λ_ex_ = 490 nm, λ_em_ = 516 nm) were excited using a blue argon laser (λ_ex_ = 488 nm), and emissions were collected at λ = 600–650 nm for the ruthenium complex and λ = 500–550 nm for Mitotracker Green. Image data acquisition and processing was performed using Olympus FV31S-SW software. The luminescence intensity of *cis*-α-[Ru(Me_4_phen)(bb_7_)]^2+^ inside the BHK cells was quantified using the surface rendering function of Imaris 9.2.1 software, and the data analyzed for statistical difference using the Wilcoxon-test and *t*-test methods.

## Results

### Toxicity against eukaryotic cells

The toxicities of the [Ru(phen′)(bb_n_)]^2+^ complexes against three eukaryotic cell lines {baby hamster kidney (BHK), human epithelial colorectal adenocarcinoma (Caco-2) and human hepatocellular carcinoma (Hep-G2)} were determined and compared to the values obtained for the dinuclear complex Rubb_12_, [Ru(Me_4_phen)_3_]^2+^ {designated (Me_4_phen)_3_, the most active of the mononuclear complexes originally identified by Dwyer and co-workers} and the control anticancer agent cisplatin. The BHK and Hep-G2 cell lines were chosen so that the IC_50_ results could be compared to those from our previous studies with multinuclear ruthenium complexes (Li et al., 2015b). It was also of interest to examine the toxicity of the ruthenium complexes against a third eukaryotic cell line that was distinct from kidney or liver cells—consequently, the Caco-2 cell line was also used in this study. The results are summarized in Table 1. The IC_50_ values determined for Rubb_12_ against the BHK and Hep-G2 cell lines in this study were similar to those obtained in an earlier study (Li et al., 2015b). Of note, α-Me_4_phen-7 showed no toxicity (>400 μM) against BHK and Caco-2 cells, and very low activity (≈ 150 μM) against Hep-G2 cells for a 24-h incubation and very low (BHK and Hep-G2) or no toxicity (Caco-2) for a 48-h incubation. By contrast, α-phen-12 was (2–5)-fold more toxic than α-Me_4_phen-7, while Rubb_12_ was generally much more toxic to the eukaryotic cells than either of the mononuclear complexes.

**Table 1:** IC_50_ values (μM) of the ruthenium complexes against the BHK, Hep-G2 and Caco-2 cell lines for 24- and 48-h incubations.

**Complexes**	**BHK**		**Hep-G2**		**Caco-2**	
	**24 h**	**48 h**	**24 h**	**48 h**	**24 h**	**48 h**
α-phen-7	>400	250 ± 42	319 ± 26	312 ± 15	>400	219 ± 43
α-Me_2_phen-7	>400	250 ± 46	358 ± 18	151 ± 30	>400	>400
α-Me_4_phen-7	>400	168 ± 5	162 ±14	142 ± 8	>400	>400
α-NO_2_phen-7	>400	>400	141 ± 29	99 ± 29	121 ± 18	126 ± 6
α-phen-12	92 ± 26	85 ± 16	94 ± 13	19 ± 3	227 ± 22	177 ± 17
(Me_4_phen)_3_	157 ± 25	111 ± 23	253 ± 60	72 ± 31	220 ± 43	265 ± 13
Rubb_12_	44 ± 9	41 ± 7	55 ± 12	15 ± 5	10 ± 1	8 ± 3
Cisplatin	>400	146 ± 8	314 ± 8	12 ± 3	376 ± 41	25 ± 6
Q[10]-α-Me_4_phen-7	>400	>400	>400	>400	>400	>400

### HSA binding

**Figure 4:**
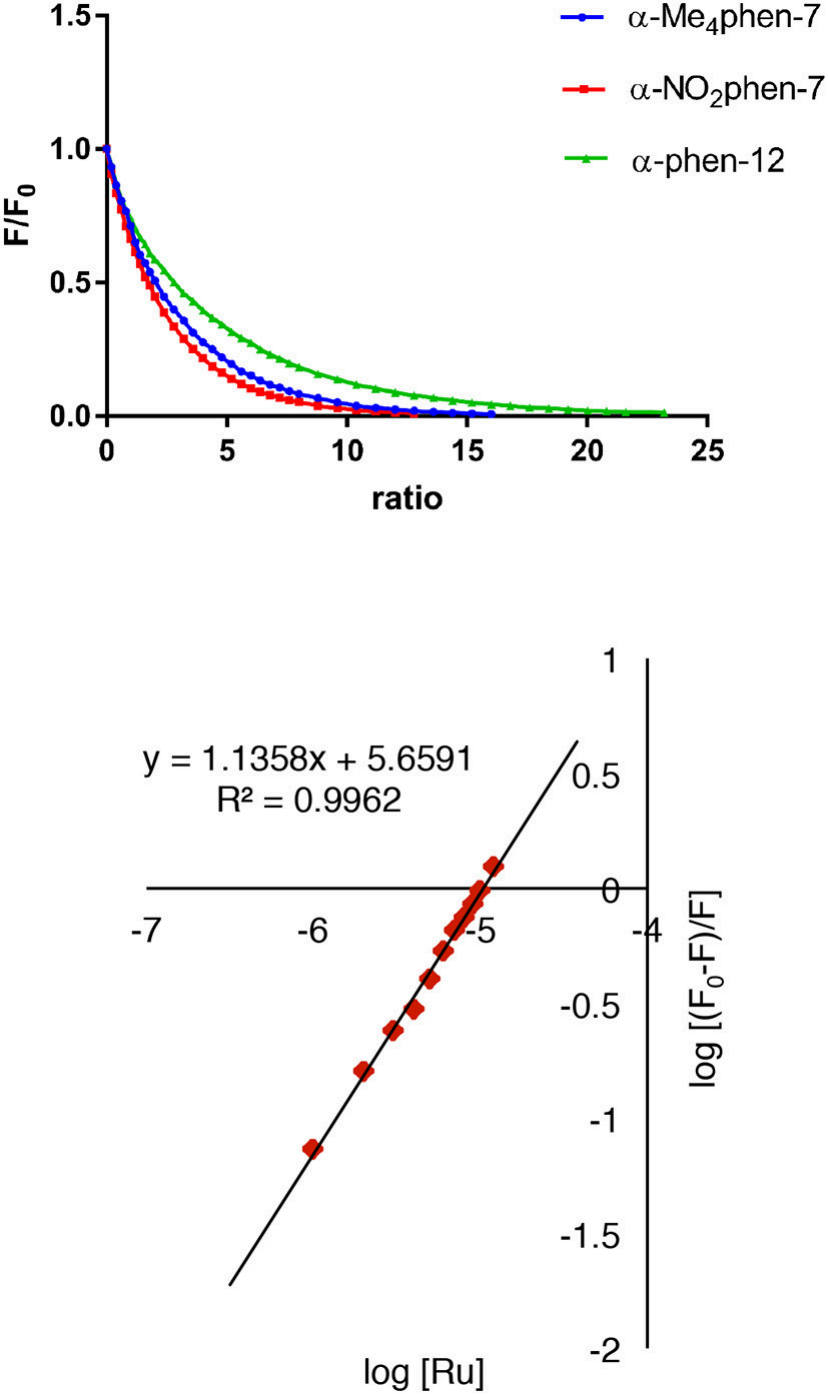
Top: relative change in the HSA fluorescence upon binding α-Me_4_phen-7, α-NO_2_phen-7 and α-phen-12; bottom: the log plot of *cis*-α-[Ru(Me_4_phen)(bb_7_)]^2+^ binding with HSA at low α-Me_4_phen-7 to HSA ratios.

The binding of the [Ru(phen′)(bb_n_)]^2+^ complexes to human serum albumin (HSA) was examined by fluorescence spectroscopy. HSA has one fluorescent tryptophan residue that can be used as a fluorophore in drug-binding experiments. The relative changes in HSA fluorescence upon binding of selected ruthenium complexes are shown in Figure 4.

All ruthenium complexes showed significant binding affinity with HSA. Consistent with a previous HSA binding study of the Rubb_n_ complexes (Li et al., 2013b), the decrease in the tryptophan fluorescence was found to be due to static quenching, a positional change of the tryptophan residue induced by the binding of the ruthenium complex to the protein.

The static quenching via binding of the ruthenium complexes to HSA can be represented as:

$$\begin{array}{l} \text{nRu}+\text{HSA}\to \text{nRu}\cdots \text{ HSA} \end{array}$$

where nRu…HSA denotes the quenched ruthenium complex/HSA species. A double-logarithm equation of log_10_[(F_0_-F)/F] vs. log_10_[Ru] can then be used to express the equilibrium between the free and bound ruthenium(II) complex to determine the apparent binding constant (K_app_) and the binding number (n) (Krause-Heuer et al., 2012).

$$\begin{array}{l} \text{log }\left[\frac{F_{0}-F}{F}\right]=\log_{10}K_{\text{app}}+\text{n}\log_{10}\left[\text{Ru}\right] \end{array}$$

It was not possible to determine a single apparent HSA binding constant, as a linear relationship was not observed over the entire concentration range of added ruthenium complex in the plot of log_10_[(F_0_ – F)/F] vs. log_10_[Ru]. However, two distinct linear sections could be found in the analysis of the double logarithm, corresponding to ruthenium complex to HSA ratios of (a) 0 to 2.4 and (b) 2.4 to 8. As previously observed (Li et al., 2013b), the two binding ratio regions represent an initial 1:1 binding at low ruthenium complex concentration (ratios 0–2.4), and then upon addition of further ruthenium complex, a region (2.4–8) where multiple ruthenium complexes (≥ 2:1) are bound to HSA. The higher affinity 1:1 HSA binding constant K_app_ and the binding numbers n for the ruthenium complexes determined at low ratios of added ruthenium complex are summarized in Table 2.

**Table 2:** The n and K_app_ values for the HSA binding of the ruthenium complexes at a [Ru complex]/[HSA] ratio from 0 to 2.4.

**Complex**	***n***	***K*_app_ × 10^5^ (M)^−1^**	**R^2^**
(Me_4_phen)_3_	0.81	0.12 ± 0.03	0.98
α-Me_4_phen-7	1.14	4.6 ± 0.90	0.996
α-Me_2_phen-7	0.91	0.29 ± 0.13	0.984
α-phen-7	0.80	0.10 ± 0.00	0.991
α-NO_2_phen-7	1.27	23.5 ± 4.9	0.998
α-phen-12	0.95	0.43 ± 0.17	0.993

For the [Ru(phen′)(bb_7_)]^2+^ complexes, the HSA binding affinity increased with the number of methyl substituents on the 1,10-phenanthroline ligand; however, the α-NO_2_phen-bb_7_ complex showed the highest binding affinity. Interestingly, the α-Me_4_phen-7 complex bound HSA with significantly higher affinity than the [Ru(Me_4_phen)_3_]^2+^ and α-phen-bb_12_ complexes, and with approximately the same affinity as previously determined for Rubb_12_ (K_app_ = 4.7 × 10^5^ M^−1^) (Li et al., 2013b).

### Q[10] encapsulation of α-me_4_phen-7

#### NMR studies

**Figure 5:**
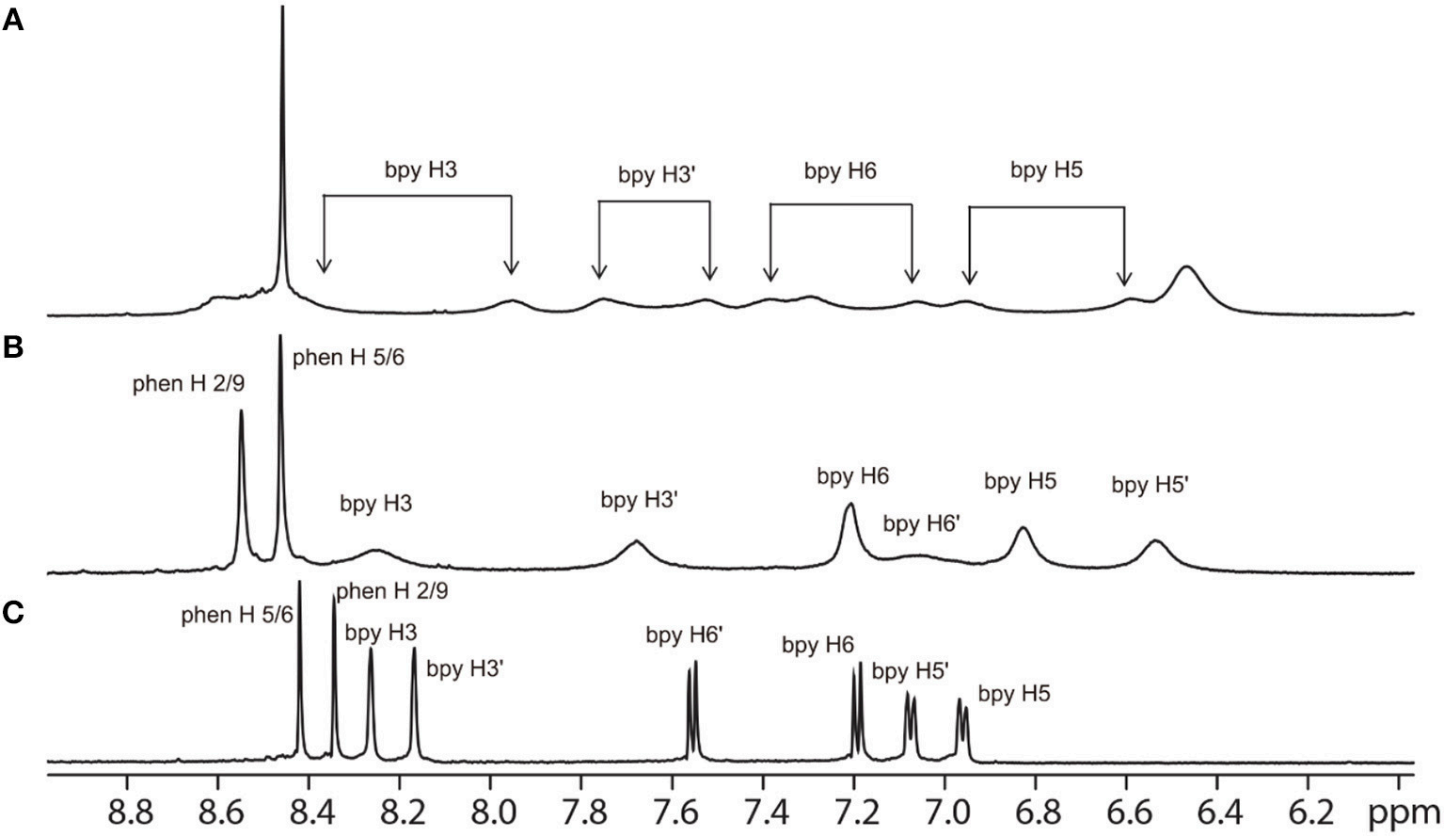
^1^H NMR spectrum of the aromatic region of the free α-Me_4_phen-7 **(C)** and with added Q[10], at Q[10] to α-Me_4_phen-7 ratios of 0.75 **(B)** and 1.0 **(A)**.

As α-Me_4_phen-7 exhibited the best differential (≈ 20-fold) between high antimicrobial activity (previously demonstrated in Sun et al., 2018) and low toxicity, it was selected for the Q[10] binding studies. Figure 5 shows the ^1^H NMR spectrum of α-Me_4_phen-7 and the spectra of the metal complex with added Q[10] at various Q[10] to α-Me_4_phen-7 ratios (R). The ^1^H NMR resonances of α-Me_4_phen-7 and the Q[10]-bound ruthenium complex were assigned from a combination of DQFCOSY and ROESY experiments. In particular, the resonances from the bb_7_ ligand were assigned through the observation of an ROE from the spin-coupled bpy H5 and H5' protons (coupled to the H6 and H6' protons) to the substituted-bpy ligand methyl (Me) and chain methylene protons respectively. The bpy H3 and H3' were similarly assigned from the observed ROEs to the bpy Me and α-CH_2_ protons, respectively. The phenanthroline H2/9 resonances were assigned through the observation of an ROE to the bb_7_ H6 protons.

One set of exchange-broadened resonances was observed for the protons from both α-Me_4_phen-7 and Q[10], indicating intermediate-exchange kinetics on the NMR timescale at 25°C for *R* < 1. Of note, the resonances from the CH_2_ groups in the alkyl chain of the bb_7_ ligand shifted significantly upfield (0.3–0.5 ppm). This indicates the alkyl chain is positioned deep within the Q[10] cavity, as many previous studies have established that resonances from guest protons located inside a Q[*n*] cavity shift significantly upfield, with protons positioned toward the middle of the Q[*n*] cavity exhibiting the largest (up to 1 ppm) upfield shifts (Mock and Shih, 1986; Jeon et al., 1996). This suggests that α-Me_4_phen-7 binds Q[10] with the bb_7_ ligand deep within the cavity and the Me_4_phen ligand projecting out of the portal. Consistent with this proposal are the significant upfield shifts (≈ 0.5 ppm) for the bb_7_ protons on the pyridyl ring containing the alkyl chain (H3', H5' and H6') coupled with the observed downfield shifts of the Me_4_phen H2/9 and H5/6 resonances.

**Figure 6:**
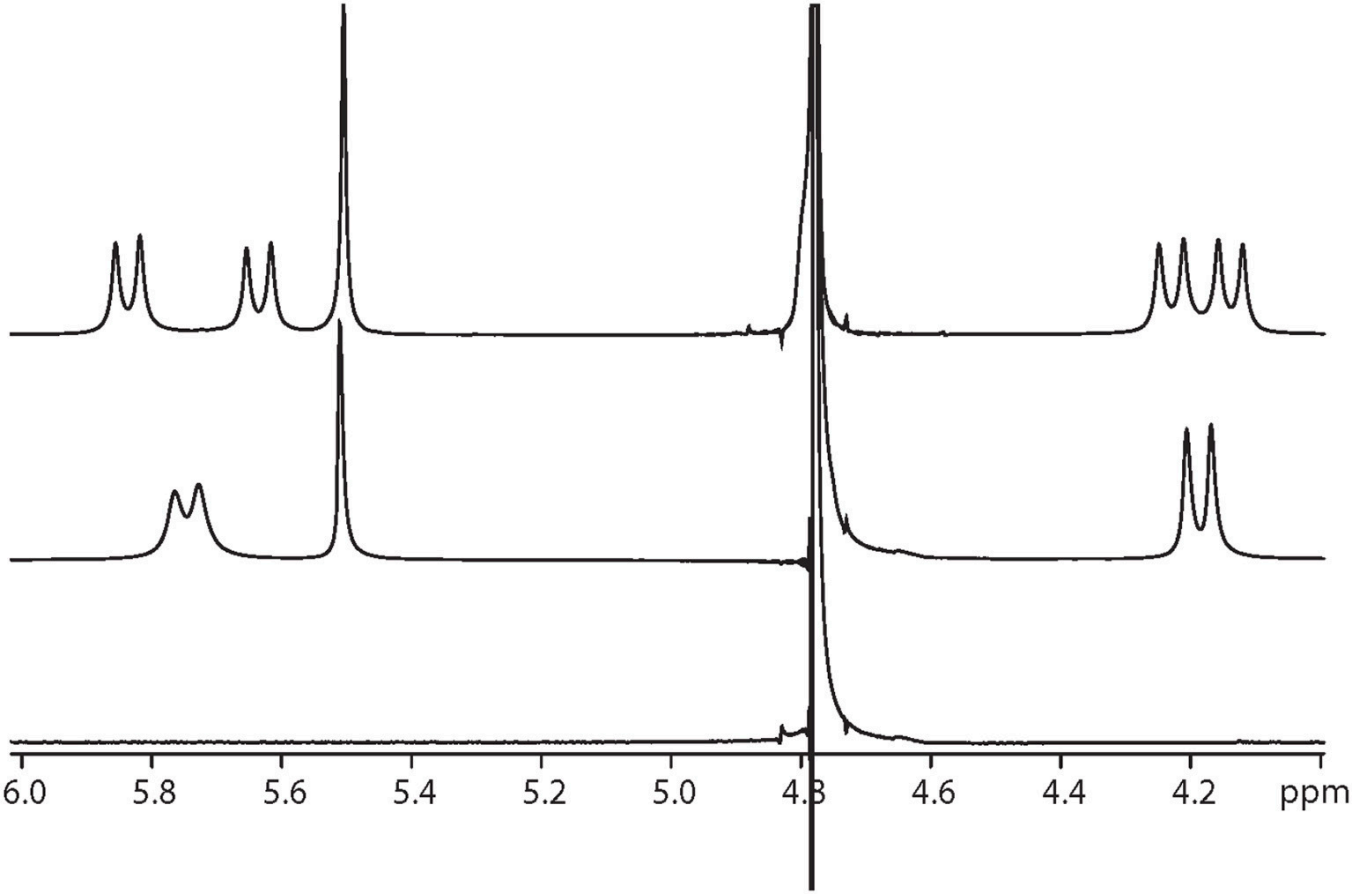
^1^H NMR spectrum of the protons from Q[10], at Q[10] to α-Me_4_phen-7 ratios of 0 (bottom spectrum), 0.75 (middle) and 1.0 (top).

Interestingly, at a Q[10] to α-Me_4_phen-7 ratio of 1 (as determined from the integration of the respective resonances), two sets of resonances for the bb_7_ aromatic protons and the Q[10] CH_2_ resonances were observed (see Figures 5, 6), indicating slow-exchange binding kinetics. The inequivalence of the Q[10] methylene resonances that project toward the portals (5.83 and 5.64 ppm) and toward the cavity center (4.23 and 4.14 ppm) is due to the non-symmetric encapsulation of α-Me_4_phen-7 in Q[10]. As intermediate-exchange kinetics were observed at *R* < 1 but slow-exchange at *R* = 1, it is concluded that for all values of *R* < 1 there must be some 1:2 (Q[10] to α-Me_4_phen-7) binding with the rate of exchange between the 1:1 and 1:2 binding modes being in the fast-exchange regime. In the *R* = 1 spectrum, the resonances from the bpy CH_3_ groups were also inequivalent, with one peak shifted downfield and the other upfield compared to equivalent bpy CH_3_ resonances in the free ruthenium complex. This suggests that one bpy CH_3_ is positioned inside the Q[10] cavity with the second bpy CH_3_ located outside of the portal. Possibly due to the broadness of the resonances from α-Me_4_phen-7, very few intermolecular ROEs were observed in ROESY spectra of the Q[10]-encapsulated ruthenium complex. However, ROEs was observed from the Me_4_phen H2/9 and 3/8 methyl resonances to the Q[10] methylene protons that project toward the portal.

#### Molecular modeling

**Figure 7:**
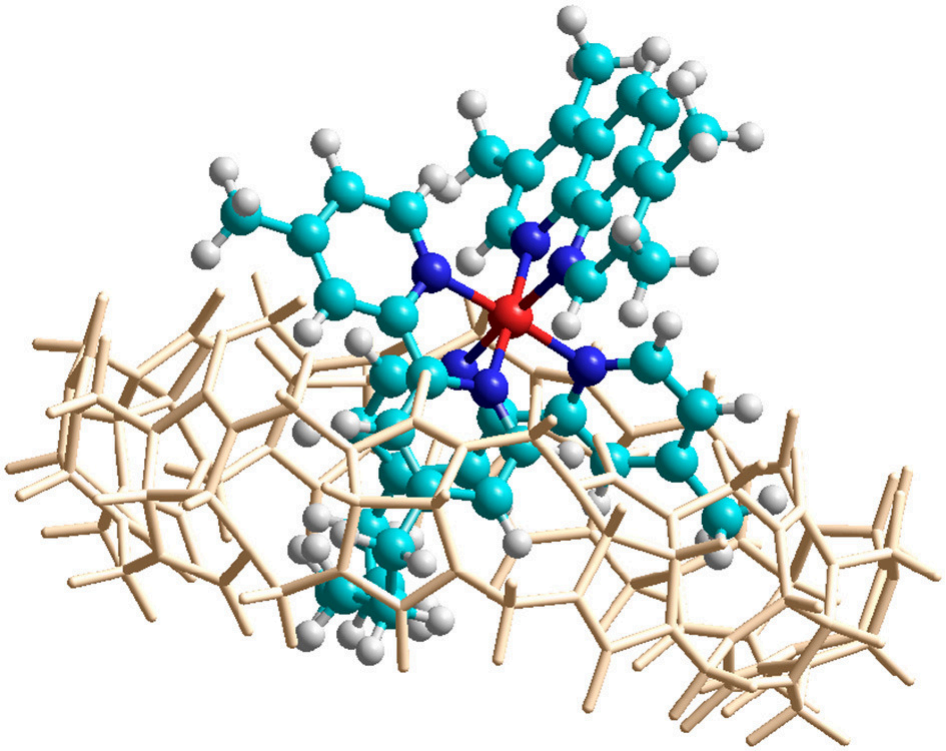
Molecular modeling of the encapsulation of α-Me_4_phen-7 with Q[10] using *HyperChem* software. The color of the atoms of α-Me_4_phen-7 are: H, white; C, cyan; N, blue; and Ru, red.

In order to confirm the feasibility of the mode of binding suggested by the NMR data, the encapsulation of α-Me_4_phen-7 within the Q[10] cavity was examined by molecular simulations using the *HyperChem* modeling software. The methylene chain of α-Me_4_phen-7 was folded within the Q[10] cavity and the ruthenium metal center positioned at the portal in a manner consistent with the observed intermolecular ROEs observed in ROESY spectra. The system was then successively optimized using the Amber99 molecular mechanics forcefield. As shown in Figure 7, stable low energy conformations could be obtained with the methylene chain folded in the cavity and the ruthenium metal center positioned outside the portal consistent with the NMR data.

#### Luminescence spectroscopy

**Figure 8:**
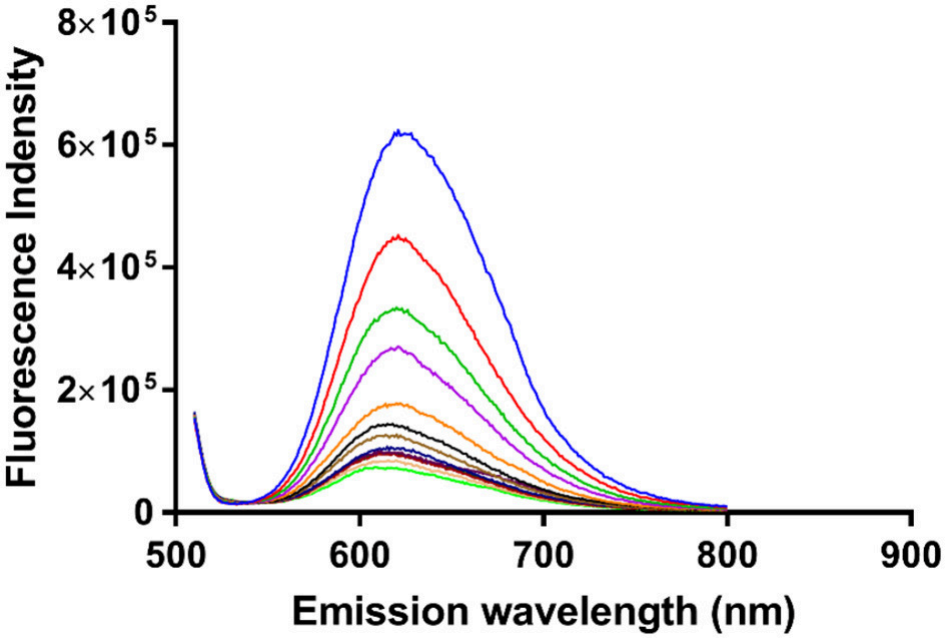
The luminescence decrease of α-Me_4_phen-bb_7_ upon the addition of Q[10] to a Q[10] to α-Me_4_phen-7 ratio of 1.5.

In order to obtain an equilibrium binding constant, the association of α-Me_4_phen-7 with Q[10] was studied by luminescence spectroscopy. The luminescence intensity of α-Me_4_phen-7 decreased upon the addition of each aliquot of Q[10] up to *R* = 1.5 (see Figure 8). Binding curves were generated by plotting luminescence intensity (counts per second) against the molar ratio of added Q[10], from which a 1:1 binding constant of 9.9 ± 0.2 × 10^6^ M^−1^ was determined.

#### Biological properties of α-me_4_phen-7 encapsulated in Q[10]

**Figure 9:**
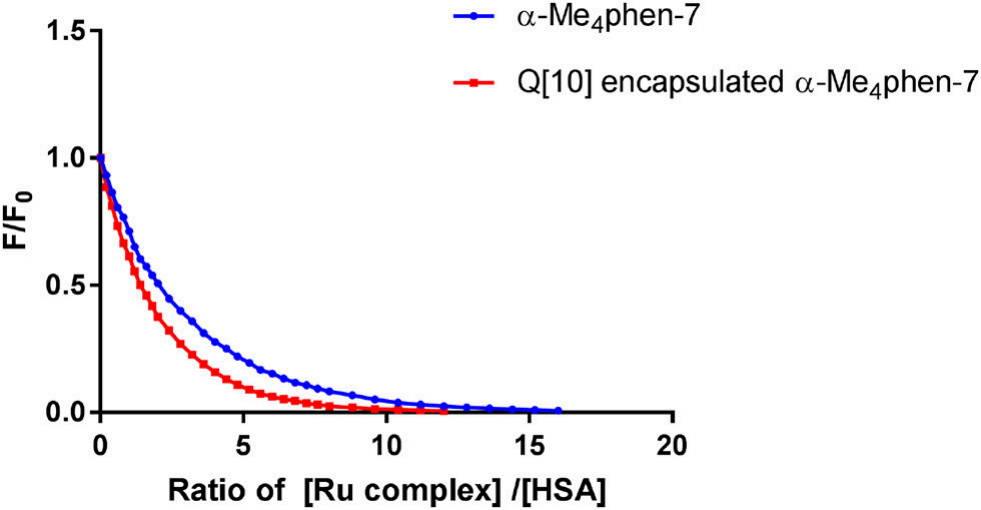
Relative change in the HSA fluorescence upon binding α-Me_4_phen-7 and α-Me_4_phen-7 encapsulated in Q[10].

Upon encapsulation in Q[10], α-Me_4_phen-7 exhibited no toxicity to any of the cell lines for both 24- or 48-h incubations (see Table 1). Based upon the 48-h incubation toxicity to BHK and Hep-G2 cells, it can be concluded that Q[10]-encapsulation decreases the toxicity of α-Me_4_phen-7 by at least (2–3)-fold. Of note, and contrary to what was observed for Rubb_12_, Q[10]-encapsulation increases the affinity of α-Me_4_phen-7 for HSA (see Figure 9). A K_app_ of 2 ± 0.2 × 10^6^ M^−1^ was determined for the Q[10]-encapsulated ruthenium complex.

**Figure 10:**
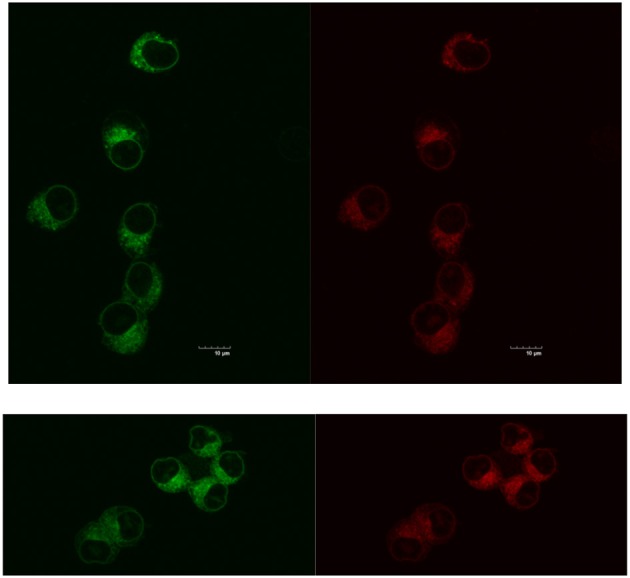
Top, left to right: α-Me_4_phen-7 accumulation in BHK cells at 25 μM after a 1-h incubation, stained by Mitotracker Green and α-Me_4_phen-7 (red). Bottom, left to right: Q[10]-encapsulated α-Me_4_phen-7 accumulation in BHK cells at 25 μM after a 1-h incubation, stained by Mitotracker Green and α-Me_4_phen-7 (red). Scale bar = 10 μm.

**Figure 11:**
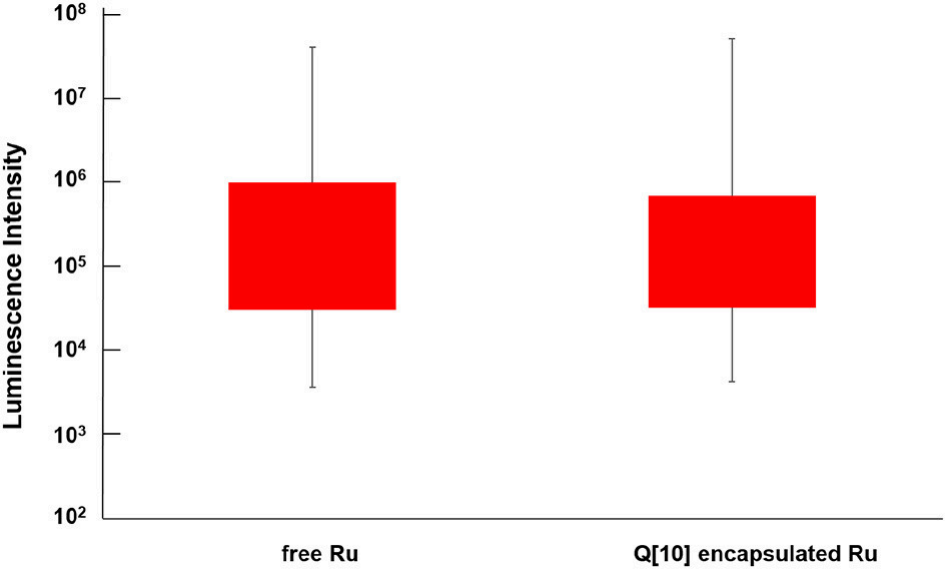
“Box and whisker” plot of the luminescence from α-Me_4_phen-7 accumulation in BHK cells at 25 μM after a 1-h incubation for both free and Q[10]-encapsulated α-Me_4_phen-7. The red box region shows the 25–75% quartiles for the luminescence intensity per cell, while the whiskers show the maximum and minimum intensities per cell.

Reducing the cellular accumulation of α-Me_4_phen-7 is the simplest mechanism by which Q[10]-encapsulation could reduce toxicity. Consequently, confocal microscopy was used to obtain both a qualitative and quantitative measure of the effect of Q[10]-encapsulation on the cellular accumulation of α-Me_4_phen-7 in BHK cells after a short incubation time. Figure 10 shows confocal microscopy images of BHK cells after a 1 h incubation with free or Q[10]-encapsulated α-Me_4_phen-7. Mitotracker Green (which selectively localizes in the mitochondria) was used to visualize the cells, with the nucleus of the cell being the dark circular region outlined by the green luminescence. As it appeared that the cellular accumulation was not significantly different between the free or Q[10]-encapsulated α-Me_4_phen-7 samples, the luminescence intensity from α-Me_4_phen-7 per cell was quantified over all the images taken. The results are shown in Figure 11. As seen in the “box and whiskers” diagram, and confirmed by statistical analysis (Wilcoxon-test and a *t*-test; *p* = 0.13 and 0.11, respectively), there was no difference in the level of the cellular accumulation of α-Me_4_phen-7 between the experiments where the ruthenium complex was added to the BHK cells in either the free or Q[10]-encapsulated forms.

## Discussion

We have previously shown that some mononuclear ruthenium(II) complexes containing bb_n_ as a tetradentate ligand (particularly α-phen-12 and α-Me_4_phen-7) exhibit good antimicrobial activity (MICs = 1–10 μM) against both Gram-positive and Gram-negative bacteria (Gorle et al., 2015; Sun et al., 2018). In this study we have examined their associated toxicity to eukaryotic cells. Importantly, although α-Me_4_phen-7 was as active against Gram-negative bacteria as α-phen-12 (Sun et al., 2018), the results from the present study showed that α-Me_4_phen-7 was (2–5)-fold less toxic to eukaryotic cells than α-phen-12. It was also previously shown that α-phen-12 (log *P* = −0.9) accumulated in bacterial cells more rapidly and to a greater degree than did α-Me_4_phen-7 (log *P* = −1.33), consistent with their respective lipophilicities (Sun et al., 2018). Consequently, α-Me_4_phen-7 appears to be more toxic to Gram-negative bacteria than α-phen-12 on a molar basis. This could be due to the greater DNA binding ability of α-Me_4_phen-7, compared to α-phen-12 (Sun et al., 2018). Of relevance in the present study, the preliminary confocal microscopy experiments indicated that α-Me_4_phen-7 did not localize in the nucleus of BHK cells. As a consequence, α-Me_4_phen-7 might not be inherently more toxic to eukaryotic cells than α-phen-12. Hence, based upon lipophilicity, it would be expected that α-phen-12 would also accumulate to greater degree in eukaryotic cells than α-Me_4_phen-7, and thereby be more toxic. It was also noted that α-Me_4_phen-7 was significantly less toxic than the lead oligonuclear complex, Rubb_12_. In absolute terms, α-Me_4_phen-7 showed very little toxicity to the eukaryotic cells and was found to be at least 20-fold more active against Gram-negative bacteria (Sun et al., 2018) than eukaryotic cells over a 24 h time period.

In terms of HSA binding, α-Me_4_phen-7 bound the serum protein approximately 10-fold more tightly than α-phen-12 and α-Me_2_phen-7, and with similar affinity as Rubb_12_. Previous studies have demonstrated that there are a number of factors governing the binding affinity of drugs with HSA (Kratochwil et al., 2002; Bohnert and Gan, 2013; Liu et al., 2014). Lipophilicity is generally considered to be important, particularly within a series of structurally-related compounds. In this study it was found the degree of methylation of the phenanthroline ligand, and hence lipophilicity, did correlate with stronger binding. However, the α-NO_2_phen-7 complex (log *P* = −1.58) bound HSA with higher affinity than α-Me_4_phen-7 (log *P* = −1.33). This observation suggests that the dispersion of the 2+ charge of the ruthenium complex is also important, which is consistent with the approximate charge of HSA at physiological pH being −17 (Fogh-Andersen et al., 1993).

It is often assumed that low serum protein binding is a “good” drug property, as it increases the concentration of the free drug; however, in a recent survey of newly approved drugs, Liu and co-workers (Liu et al., 2014) found that 45 and 24% of the drugs exhibited high (>95% bound) or very high (>99%) serum protein binding, respectively. Consequently, Liu et al and others proposed that serum protein binding is neither an intrinsically “good” or “bad” property. However, a recent pharmacokinetic study of Rubb_12_ in mice demonstrated that the amount of the ruthenium complex in the serum decreased very rapidly after administration by intravenous injection (Li et al., 2017). This result suggests that potential new ruthenium complexes would need to bind serum proteins at least as strongly, if not with greater affinity, than Rubb_12_. Based upon this criterion, it is noted that α-Me_4_phen-7 binds HSA with at least similar affinity to Rubb_12_.

Given the antimicrobial potential of α-Me_4_phen-7, it was of interest to examine the ability of the ruthenium complex to bind Q[10], and determine its subsequent effect on cytotoxicity and HSA binding. The results of this study showed that α-Me_4_phen-7 does form a high-affinity water-soluble association complex with Q[10], with the bb_7_ alkyl chain positioned deep within the cavity and the ruthenium metal center positioned at one of the portals. This binding mode, with the non-polar segment of a metal complex in the hydrophobic cavity and the cationic metal center positioned at the carbonyl-rimmed hydrophilic portals, is consistent with previous studies (Pisani et al., 2010; Alrawashdeh et al., 2016). The magnitude of the α-Me_4_phen-7-Q[10] binding constant (≈10^7^ M^−1^) indicates that a large proportion of the ruthenium complex would be Q[10]-bound at the micro-molar concentrations that would be found in the blood after administration.

The effect of Q[*n*] encapsulation on the cytotoxicity of a variety of compounds toward eukaryotic cell lines has been previously examined. For example, Kim et al. reported a (5–10)-fold decrease in activity for oxaliplatin in Q[7] (Jeon et al., 2005), while Wheate et al showed that a dinuclear platinum complex maintained its activity toward L1210 cells, but was 2-fold less active against the cisplatin resistant cell line L1210/DDP (Wheate et al., 2004). Furthermore, there have been several recent reports where encapsulation in Q[*n*] was proposed to have increased the uptake of a cytotoxic agent (Konda et al., 2017; Shinde et al., 2018). In the present study, Q[10]-encapsulation decreased the toxicity of α-Me_4_phen-7 toward BHK and Hep-G2 cells, while no conclusions could be drawn for the Caco-2 cells, given the lack of activity of the free ruthenium complex for both 24- and 48-h incubations. The most obvious explanation for the decreased toxicity of α-Me_4_phen-7 encapsulated in Q[10] is that the concentration of the free, unbound, ruthenium complex is significantly lower in the assay medium and the Q[10]-bound ruthenium complex can not cross the cell membrane. This would consequently result in a slower and lower accumulation of the ruthenium complex inside the cells. However, the results from the confocal microscopy experiments indicated that the level of accumulation of α-Me_4_phen-7 inside the cells is not significantly affected by encapsulation over a 1-h incubation.

In terms of HSA binding, Q[10]-encapsulation increased the affinity of α-Me_4_phen-7 for the serum protein. Interestingly, in an earlier study it was determined that Q[10]-binding of Rubb_12_ decreased the affinity of the ruthenium complex for HSA binding (Li et al., 2013b). As noted above, given the short residence time found for Rubb_12_ after intravenous injection in mice, the (3–4)-fold higher HSA binding affinity is likely to be beneficial.

## Conclusions

In conclusion, the results of this study have demonstrated that the most active antimicrobial mononuclear ruthenium complex against Gram-negative bacteria, α-Me_4_phen-7, is also the least toxic to eukaryotic cells. Furthermore, although it exhibits slightly lower (≈ 2-fold) antimicrobial activities to Gram-negative bacteria than the lead oligonuclear complex Rubb_12_ (Sun et al., 2018), α-Me_4_phen-7 is also significantly less toxic to eukaryotic cells compared to the dinuclear complex. Fluorescence assays demonstrated that α-Me_4_phen-7 bound HSA with similar affinity to that previously reported for Rubb_12_. Taken together, the results of this study suggest α-Me_4_phen-7 is a suitable candidate for further studies of its antimicrobial properties. In addition, it has been shown that α-Me_4_phen-7 can form a high-affinity, water-soluble, inclusion complex with Q[10]. Q[10]-encapsulation decreases the toxicity of α-Me_4_phen-7 toward eukaryotic cells and increases its HSA binding affinity. As a consequence, administration of α-Me_4_phen-7 encapsulated in Q[10] may increase the potential of the ruthenium complex as an antimicrobial agent. However, the effect of Q[10]-encapsulation on the antimicrobial properties of α-Me_4_phen-7 needs to be examined.

## Author contributions

BS synthesized the ruthenium complexes and carried out the experiments. AD provided the Q[10], while IM, KH, FK, and JC were involved in planning the research, analysing the data, and writing the paper.

### Conflict of interest statement

The authors declare that the research was conducted in the absence of any commercial or financial relationships that could be construed as a potential conflict of interest.
